# Profile of forensic medical examinations among members of the Battalion of Special Police Operations of Santa Catarina, Brazil

**DOI:** 10.47626/1679-4435-2023-1180

**Published:** 2024-11-14

**Authors:** Leonardo Duarte Soares, João Pedro dos Reis Morfim, Ariana Lebsa Weber, Flavio Ricardo Magajewski, Jefferson Traebert

**Affiliations:** 1 Medical School, Universidade do Sul de Santa Catarina (Unisul), Palhoça, SC, Brazil; 2 Directorate of Health and Social Promotion, Polícia Militar de Santa Catarina, Florianópolis, SC, Brazil; 3 Graduate Program in Health Sciences, Unisul, Palhoça, SC, Brazil

**Keywords:** police, occupational diseases, accidents, occupational, epidemiology, polícia, doenças profissionais, acidentes de trabalho, epidemiologia

## Abstract

**Introduction:**

The military police officer is exposed to several risk factors that could lead to physical and psychiatric disorders. However, there is a paucity of scientific literature on the health of special operations police officers.

**Objectives:**

To identify the main causes and associated factors of forensic medical examinations among members of a Battalion of Special Police Operations.

**Methods:**

This cross-sectional study analyzed data from 210 forensic medical examinations performed between January 2019 and June 2021. The dependent variable was the reason for the appointment, and the independent variables were gender, age, military rank, and length of career. Bivariate analyses were performed using the chi-squared test or Fisher’s exact test.

**Results:**

Most police officers were men (98.1%), military (41.8%), with a mean age of 36.6 years and a mean length of service of 12.9 years. The most common reason for an examination was to obtain a physical fitness report (44.3%), followed by routine examination of healthy police officers (17.6%) and trauma (14.3%). There were no statistically significant associations between the independent variables and trauma appointments. However, there was a statistical association between appointments for physical fitness reports and being under 35 years of age.

**Conclusions:**

Most common reasons for forensic medical examinations were physical fitness reports, routine examinations, and trauma. Examinations for physical fitness reports were statistically associated with younger age.

## Abstract (pt)

**Introdução:**

O policial militar está exposto a diversos fatores de risco que o predispõem a doenças físicas e psiquiátricas. No entanto, a literatura científica é limitada em relação à saúde de policiais que atuam em operações policiais especiais.

**Objetivos:**

Conhecer os principais motivos e fatores associados a consultas médicas periciais em membros de um Batalhão de Operações Policiais Especiais.

**Métodos:**

Estudo transversal envolvendo dados de prontuários de 210 consultas periciais realizadas por 66 policiais militares entre janeiro de 2019 e junho de 2021. A variável dependente foi o motivo de consulta, e as independentes foram sexo, idade, graduação e tempo de carreira. A análise bivariada foi realizada por meio do teste do qui-quadrado ou prova exata de Fisher.

**Resultados:**

A maioria dos pacientes era representada por homens (98,1%), soldados (41,8%) com idade média de 36,6 anos e tempo médio de carreira de 12,9 anos. O motivo de consulta mais prevalente foi de emissão de laudo de aptidão física (44,3%), seguido de exames periódicos em paciente hígido (17,6%) e trauma (14,3%). Não foram observadas associações estatisticamente significativas entre as variáveis independentes e o motivo de consulta por trauma, porém houve associação estatística entre consulta por emissão de laudo de aptidão física e idade menor que 35 anos.

**Conclusões:**

Os principais motivos de consulta foram por emissão de laudo de aptidão física, exames periódicos e trauma. O motivo de consulta por emissão de laudo de aptidão física mostrou-se associado à idade.

## INTRODUCTION

The Brazilian Battalion of Special Police Operations (BOPE) is a highly specialized police force trained to deal with situations such as hostage rescue, armed suicides, incidents involving explosive devices, and fighting criminal gangs, etc. Current scientific literature lacks data on this occupational category, but evidence suggests that police officers in general are at higher risk for musculoskeletal disorders and psychiatric conditions.^1^ The military police profession is fraught with stressors such as excessive working hours and lack of rest, with many officers having at least one additional employment relationship outside their primary institution.^2^

In addition, subjective factors such as a negative perception of job value, low career growth prospects, and dissatisfaction with service compensation negatively affect quality of life.^3^ These factors are directly related to negative psychological outcomes.^4^ A significant number of police officers may screen positive for depression, anxiety, and posttraumatic stress disorder.^5^ In addition, police officers have a 54% higher risk of suicide compared to other occupations.^6^

Despite the intense physical demands of the police profession, literature also highlights significant health risk factors such as overweight and obesity, physical inactivity, smoking, and alcohol consumption,^7^ which negatively impact job performance. Regarding musculoskeletal disorders, operational training and encounters with aggressive suspects are thought to be the main causes of such injuries, with sprains and strains being the most common.^8^

Lovalekar et al.^9^ showed Naval Special Warfare (NSW), a specialized unit of the U.S. Navy, incurred nearly $3 million in musculoskeletal injuries over the course of 1 year. One-third of this amount was attributed to potentially preventable injuries. Another study of members of the United States Air Force Special Operations Command (AFSOC), another U.S. special operations force, identified a musculoskeletal burden of over $1 million, with just over one-third of that amount attributed to potentially preventable injuries.^10^

Despite studies performed with other sectors of the Brazilian Military Police, national literature lacks studies on the profile of forensic examinations in the context of special police operations. Forensic medical examinations are characterized by the medical act performed by an isolated forensic physician, a professional legally qualified for forensic activity, which consists in the technical evaluation of health issues related to work capacity, fitness for physical activity, and participation in operational courses.^11^ Thus, the present study aimed to identify the main reasons and factors associated with forensic examinations among members of a BOPE.

Answering this research question may help to reduce the burden of health care needs as well as provide a better understanding of injuries and lost work time in police officers and contribute to their improvement.

## METHODS

The present study was designed as cross-sectional research. It uses data from members of the Sanitary Formation of the 11th Military Police Region of the Military Police of Santa Catarina (PMSC). This unit is responsible for the state police forces stationed in the cities of São José, Biguaçu, Palhoça, Governador Celso Ramos, Santo Amaro da Imperatriz, Águas Mornas, and Paulo Lopes.

At the time of this study, BOPE of Santa Catarina had 70 members. Study population consisted of data from 66 members who required a forensic medical examination between January 2019 and June 2021.

Inclusion criteria for this study were to be a member of the BOPE of Santa Catarina, and to have attended a forensic medical examination between January 2019 and June 2021. Since data were processed in a computerized system independent of physical medical records, the sources of this study, consultations related to the COVID-19 health emergency were not included in this research. In addition, follow-up consultations were excluded from the data analysis in order to obtain a more accurate analysis of the reasons for consultations.

Data collection was done by consulting the medical records with the written authorization of their custodian. The dependent variable considered was the reason for the consultation (issuance of a physical fitness certificate, periodic examinations in healthy patients, trauma, other reasons, no reason given). The independent variables were: sex (male/female), age range in years according to the median distribution (up to 35 years/35 years or older), rank (soldier and corporal/sergeant and officers), and length of service in the BOPE in years according to the median distribution (up to 13 years/13 years or more).

Data were entered into Excel spreadsheets and later exported to IBM SPSS 18.0 for analysis. Descriptive statistics were performed on the variables studied. Bivariate analysis between the dependent variable and the independent variables was performed using the chi-squared test or Fisher’s exact test, with significance considered at p < 0.05.

The study was submitted to and approved by the Research Ethics Committee at Universidade do Sul de Santa Catarina, with opinion number 5.175.765. The use of the Informed Consent Form was waived, as the work was performed using medical record data from patients who were not undergoing treatment at the time.

## RESULTS

Data from 210 forensic examinations performed at the medical service of the Sanitary Formation of the 11th Region of the PMSC from January 2019 to June 2021 were included in this study. Most police officers were men (98.1%), with a mean age of 36.6 years (SD = 5.9). The most common rank was soldier, with 41.9%, and the mean length of service was 12.9 years (SD = 7.1) (Table 1).

**Table 1:** Characteristics of the study population, Battalion of Special Police Operations (BOPE), Sanitary Formation of the 11th Region of the Military Police of Santa Catarina (PMSC), Greater Florianópolis, January 2019 to June 2021 (n = 210)

Variable	n (%)	Mean (SD)
Sex		
Male	206 (98.1)	
Female	4 (1.9)	
Rank		
Soldier	88 (41.8)	
Corporal	34 (16.2)	
Sergeant	75 (35.7)	
1st Lieutenant	1 (0.5)	
Captain	6 (2.9)	
Major	6 (2.9)	
Age (years)		36.6 (5.9)
Length of service (years)		12.9 (7.1)

SD = standard deviation

Issuing a fitness certificate was the most common reason for all forensic examinations. The reasons are shown in Table 2.

**Table 2:** Reasons for forensic examinations, Battalion of Special Police Operations (BOPE), Sanitary Formation of the 11th Region of the Military Police of Santa Catarina (PMSC), Greater Florianópolis, January 2019 to June 2021 (n = 210)

Reasons for examinations	n (%)
Healthy patients	139 (66.2)
Physical fitness certificate	93 (44.3)
Periodic examinations	37 (17.6)
Engagement / reengagement	9 (4.3)
Consultations for health issues	59 (28.6)
Trauma	30 (14.3)
Gastroenteritis	4 (1.9)
Family member consultation	3 (1.4)
Urinary tract calculi	2 (1.0)
Mucopurulent conjunctivitis	2 (1.0)
Course fitness certificate (unspecified)	2 (1.0)
Acute nasopharyngitis	2 (1.0)
Adverse reaction to viral vaccine	1 (0.5)
Unspecified adverse reaction	1 (0.5)
Verification of origin certificate	1 (0.5)
Cutaneous abscess, furuncle	1 (0.5)
Unilateral blindness	1 (0.5)
Screening examination for intestinal tract neoplasia	1 (0.5)
Intestinal malabsorption	1 (0.5)
Acute myocarditis	1 (0.5)
Obesity due to excess calories	1 (0.5)
Otalgia	1 (0.5)
External otitis	1 (0.5)
Nonsuppurative otitis media	1 (0.5)
Bacterial pneumonia	1 (0.5)
Post-cataract	1 (0.5)
No reason reported	12 (5.7)

Trauma was the most common reason for forensic examinations among unhealthy police officers. Table 3 shows descriptive analysis of type, location, precipitating activity, and service situation for trauma cases.

**Table 3:** Descriptive analysis of trauma cases, Battalion of Special Police Operations (BOPE), Sanitary Formation of the 11th Region of the Military Police of Santa Catarina (PMSC), Greater Florianópolis, January 2019 to June 2021 (n = 210)

Trauma cases	n (%)
Type	
Fracture	11 (36.7)
Sprain, strain, and contusion	11 (36.7)
Soft tissue rupture	6 (20.0)
Gunshot injuries	2 (6.7)
Location	
Lower limbs	16 (53.3)
Upper limbs	7 (23.3)
Head and neck	3 (10.0)
Unspecified	4 (13.3)
Triggering activity	
Self-defense practice	12 (40.0)
Motorcycle accident	9 (30.0)
Other physical activities	5 (16.7)
Police pursuit	2 (6.7)
Firearm training	2 (6.7)
Service situation	
On duty	17 (56.7)
Off duty	13 (43.3)

Findings of association studies are presented in Tables 4 and 5. No statistically significant associations were observed between the independent variables and the reason for consultation for trauma (Table 4). The reason for seeking a physical fitness certificate was associated with age. Individuals under 35 years of age sought more consultations for this reason than those over 35 years of age (p = 0.036) (Table 5).

**Table 4:** Forensic examinations for trauma according to the variables studied, Battalion of Special Police Operations (BOPE), Sanitary Formation of the 11th Region of the Military Police of Santa Catarina (PMSC), Greater Florianópolis, January 2019 to June 2021 (n = 210)

Variable	Trauma (n)	Others (n)	Total (n)	P-value
Sex				0.537
Male	30	176	206	
Female	-	4	4	
Age in years (median)				0.193
> 35	17	79	96	
≤ 35	13	101	114	
Rank				0.864
Soldier and corporal	17	105	122	
Sergeants and officers	13	75	88	
Career length in years (median)				0.955
> 13	14	85	99	
≤ 13	16	95	111	

**Table 5:** Forensic examinations for issuing physical fitness certificate according to the variables studied, Battalion of Special Police Operations (BOPE), Sanitary Formation of the 11th Region of the Military Police of Santa Catarina (PMSC), Greater Florianópolis, January 2019 to June 2021 (n = 210)

Variable	Physical fitness (n)	Others (n)	Total (n)	P-value
Sex				0.324
Male	90	116	206	
Female	3	1	4	
Age in years (median)				0.036
≤ 35	58	56	114	
> 35	35	61	96	
Rank				0.394
Soldier and corporal	51	71	122	
Sergeants and officers	42	46	88	
Career length in years (median)				0.608
≤ 13	51	60	111	
> 13	42	57	99	

## DISCUSSION

Data obtained in the present study showed the most common reasons for forensic examinations were the issuance of a physical fitness certificate (44.3%) and periodic examinations of healthy patients (17.6%). This indicates most consultations were performed on healthy police officers and were motivated by the requirements of the BOPE. Among the reasons related to health problems, trauma was the most common; however, no statistical associations were found between the independent variables and these reasons for consultation. A statistically significant association was observed between age and consultation for a physical fitness certificate, which suggests patients under the age of 35 may be more likely to seek consultation for this reason.

The fact that most visits were for healthy patients is consistent with data from the Military Health System (MHS),^12^ the U.S. Armed Forces health care system, which showed that in 2020, more than 40% of outpatient visits by active-duty U.S. military personnel were for non-illness related reasons, such as routine check-ups.

The results of this study support the literature by demonstrating the high level of health among special operations forces personnel, as evidenced by the high proportion of consultations with healthy patients compared to those with health problems. This was observed when the health levels of these forces were compared to those of conventional troops.^13^ Military police have a high prevalence of overweight and physical inactivity,^14^ which increases the likelihood of developing metabolic syndrome.^15^ In contrast, there was only one obesity-related consultation in the study population. A possible explanation for this difference could be the intense physical, psychological, and technical preparation required to perform special operations, which may require the daily maintenance of healthy lifestyle habits by special operations personnel.

Among health-related consultations, trauma was the most common reason, confirming data highlighting musculoskeletal disorders as the main reason for consultations in the U.S. Armed Forces^12^ and among military police in the state of São Paulo.^16^ Most of the traumas were fractures or sprains that occurred during service, mainly due to self-defense training and motorcycle accidents. Despite the dangerous nature of police, traumatic events from work-related violence were among the least common (6.7%), contrary to previous findings in the literature indicating violence as the main cause of trauma among police officers.^8^

On the other hand, self-defense training combined with other physical activities accounted for 56.7% of trauma cases, which is consistent with findings that highlight physical training as a major cause of trauma in military personnel.^17^ The high prevalence of sprains (36.7%) may be due to the intense physical training that operators undergo, with literature suggesting an association between training and sprains, particularly at the ankle and knee levels.^18^ A total of 30% of the trauma cases were caused by motorcycle accidents, which suggests they are a significant cause of morbidity among police officers, especially considering that motorcyclists are more prone to severe injuries and longer hospital stays compared to occupants of other vehicles.^19^

There were no forensic consultations for psychiatric reasons during the study period; however, the literature shows that the police population is vulnerable to psychiatric disorders in addition to physical disorders, including major depressive disorder, posttraumatic stress disorder, and generalized anxiety disorder, etc.^20^ Important risk factors for these disorders include low social support, high demands, inadequate material conditions, and low compensation, all of which are present in the reality of the Brazilian military police.^3,21^

The absence of forensic consultations for such reasons may indicate the possible presence of protective factors in the population studied, possible underreporting, or the omission of psychiatric complaints among participants. The ideal culture of physical and mental resilience within the military institution itself could justify the suppression of physical and psychiatric problems to avoid stigma from colleagues,^22,23^ especially in the case of special forces personnel.

The higher demand for forensic medical examinations by young police officers for the issuance of physical fitness certificates can be explained by the fact they are in the early stages of their police career, undergoing more rigorous training routines, or motivated to advance within the institution. This motivation leads them to participate more intensively in courses and specializations and to seek conditions for promotion and career advancement.

The results obtained in this study indicate BOPE officers have a health profile adapted to the nature of their work, which requires a high level of physical and mental health.

Physical training was confirmed as the main cause of trauma among operators, especially due to martial arts training. A possible strategy to minimize injuries from this cause would be to reinforce the correct use of protective equipment during training and to review the physical activity loads imposed on police officers in their work schedules. The low prevalence of injuries during police activities suggests that, despite the high-risk nature of the profession, police officers are well-prepared to handle such situations.

The Brazilian literature lacks studies related to the health of military police officers involved in special operations. Therefore, new studies with a specific focus on this population are pertinent, especially regarding the hypothesis of protective factors and the underreporting of psychiatric disorders. In addition, it is suggested that studies explore the extent of the burden caused by traumatic events in the preparation and maintenance of the physical condition of military personnel, which was the main health issue in the population of this study.

An important limitation of the present study is the small sample size available for analysis. This was due to the limited number of members in the BOPE unit studied, combined with the fact that the consultations analyzed occurred during the peak of the pandemic caused by severe acute respiratory syndrome coronavirus 2 (SARS-CoV-2), a period during which medical consultations in the institution were severely restricted, with some consultations being processed through a different record-keeping system. In addition, the non-standardized way in which medical records were completed made data collection and organization difficult.

## CONCLUSIONS

The main reasons for which BOPE members sought forensic medical examinations were the issuance of physical fitness certificates, routine examinations of healthy patients and traumas, the latter mainly caused during training and physical activities performed within the company. The reason for consulting a doctor for a certificate of physical fitness was related to age.
